# HBoV-1: virus structure, genomic features, life cycle, pathogenesis, epidemiology, diagnosis and clinical manifestations

**DOI:** 10.3389/fcimb.2023.1198127

**Published:** 2023-05-17

**Authors:** Mehrdad Mohammadi

**Affiliations:** ^1^ Social Security Organization, Isfahan, Iran; ^2^ Microbiology and Immunology, Faculty of Medicine, Kashan University of Medical Sciences, Kashan, Iran

**Keywords:** human bocavirus, HBoV-1, pathogenesis, epidemiology, bocaparvovirus

## Abstract

The single-stranded DNA virus known as human bocavirus 1 (HBoV-1) is an icosahedral, linear member of the *Parvoviridae* family. In 2005, it was discovered in nasopharyngeal samples taken from kids who had respiratory tract illnesses. The HBoV genome is 4.7–5.7 kb in total length. The HBoV genome comprises three open-reading frames (ORF1, ORF2, and ORF3) that express structural proteins (VP1, VP2, and VP3), viral non-coding RNA, and non-structural proteins (NS1, NS1-70, NS2, NS3, and NP1) (BocaSR). The NS1 and NP1 are crucial for viral DNA replication and are substantially conserved proteins. Replication of the HBoV-1 genome in non-dividing, polarized airway epithelial cells. *In vitro*, HBoV-1 infects human airway epithelial cells that are strongly differentiated or polarized. Young children who have HBoV-1 are at risk for developing a wide range of respiratory illnesses, such as the common cold, acute otitis media, pneumonia, and bronchiolitis. The most common clinical symptoms are wheezing, coughing, dyspnea, and rhinorrhea. After infection, HBoV-1 DNA can continue to be present in airway secretions for months. The prevalence of coinfections is considerable, and the clinical symptoms can be more severe than those linked to mono-infections. HBoV-1 is frequently detected in combination with other pathogens in various reports. The fecal-oral and respiratory pathways are more likely to be used for HBoV-1 transmission. HBoV-1 is endemic; it tends to peak in the winter and spring. This Review summarizes the knowledge on HBoV-1.

## Introduction

In 2005, the *human bocavirus 1* (HBoV-1), An autonomous human *Parvovirus* member of the family *Parvoviridae*, was found in nasopharyngeal samples taken from children suffering from respiratory tract illnesses (Allander et al., 2005). *Parvoviruses* are non-enveloped, icosahedral viruses. These are linear single-stranded DNA (ssDNA) genomes of 4.7–5.7 kilobases (Cotmore and Tattersall, 2014; Cotmore et al., 2014; Kailasan et al., 2015). The *Parvovirinae* subfamily comprises eight genera. This subfamily consists of *Amdoparvovirus*, *Aveparvovirus*, *Bocaparvovirus*, *Copiparvovirus*, *Dependoparvovirus*, *Erythro-parvovirus*, *Protoparvovirus*, and *Tetraparvovirus*. *Bocaparvoviruses* include HBoV-1, BPV, and MVC. No virus was isolated or cultivated, and only fragmentary viral sequences were found (Cotmore and Tattersall, 2014; Cotmore et al., 2014; Kailasan et al., 2015; Qiu et al., 2017).

HBoV causes severe respiratory tract infections in young infants (Christensen et al., 2019). In 2009 and 2010, researchers found three new bocaviruses designated as HBoV2–4 (Kapoor et al., 2009; Kapoor et al., 2010). The vast majority of these viruses have been discovered in feces samples, yet unknown what function they play in human illnesses (De et al., 2017). These viruses have been detected in the feces of between 1% and 40% of children with and without digestive problems (Kapoor and Dhole, 2016; La Rosa et al., 2016; Ong et al., 2016; De et al., 2017).

Bocaparvoviruses’ entire genomes, including terminal hairpins, have been sequenced. The 5,543-nt HBoV-1 genome (Genbank accession no. JQ923422) has hairpin structures at both ends (Kakizaki et al., 2022). Two temporary terminal hairpin structures border the parvovirus ssDNA genome, which is essential for viral replication. We think bocaparvoviruses replicate DNA using the rolling hairpin mechanism like other parvoviruses (Schildgen et al., 2012; Abramczuk et al., 2015).

In a laboratory setting, HBoV-1 can infect specialized human airway cells called polarized epithelial cells (Shao et al., 2021). During the infection process, the virus produces a total of ten different proteins, including six non-structural proteins (NS1, NS1-70, NS2, NS3, NS4, and NP1), a type of RNA that does not code for proteins known as BocaSR, and three structural proteins (VP1, VP2, and VP3) (Qiu et al., 2017; Christensen et al., 2019; Shao et al., 2021). These proteins play different roles in the virus’s lifecycle, including replication and assembly of new viral particles. HBoV-1 gene products enhance wild-type AAV replication in HEK 293 cells and polarized human airway epithelial cells, with high tropism observed for the HBoV-1 capsid-based rAAV vector in human airway epithelia (Mattola et al., 2022; Xie et al., 2022). Approximately 25% of children with respiratory diseases showed HBoV-1 in their airways (Malta et al., 2020). Children infected with HBoV-1 are more likely to suffer from various respiratory illnesses, including acute otitis media, pneumonia, the common cold, bronchiolitis, and asthma exacerbations (Christensen et al., 2019; Lee et al., 2019; Bagasi et al., 2020; Malta et al., 2020). After an acute infection, there may be HBoV-1 DNA in the secretions of the airways for many months (Abramczuk et al., 2015). HBoV DNA can be detected in serum, cerebrospinal fluid, urine, tonsillar tissue, tumor tissue, sewage, and river water samples (Gill et al., 2017; Christensen et al., 2019; Lee et al., 2019; Bagasi et al., 2020). As a result, traditional DNA PCR cannot be used to identify acute HBoV-1 infection; quantitative PCR and serology are superior diagnostic techniques. The frequently used HBoV-1 DNA PCR test has low clinical specificity due to the presence of HBoV-1 in nasopharyngeal aspirates from healthy infants (Neske et al., 2007; Zaghloul, 2011; Koseki et al., 2012; Bruning et al., 2016; Malta et al., 2020).

Consequently, qualitative PCR cannot alone diagnose HBoV-1 infections. Diagnostic advances such as detecting HBoV-1 mRNA and antigen have shown promising outcomes due to their excellent clinical specificity. HBoV-1, the most clinically relevant human bocavirus, should be tested in hospitalized children with respiratory tract infections. Despite its prevalence, many physicians fail to detect pediatric HBoV-1 (Neske et al., 2007; Zaghloul, 2011; Koseki et al., 2012; Bruning et al., 2016; Malta et al., 2020). This Review briefly focuses on the clinical characteristics of epidemiology, fundamental virology, and pathophysiology and diagnostic implications of HBoV-1.

## Genomic features and virus structure of HBoV-1

**Figure 1:**
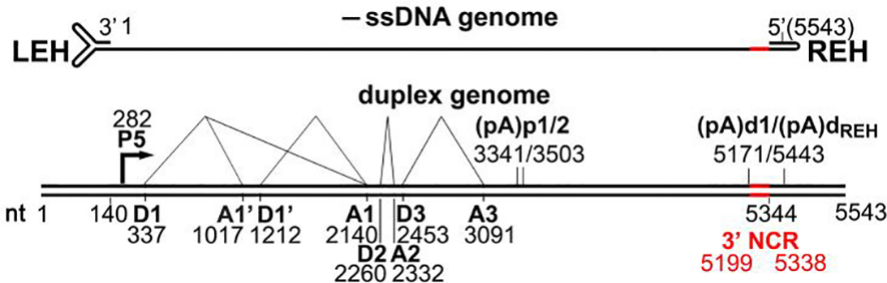
Genomic structure of HBoV-1.

*Parvoviruses* are non-enveloped, extremely small (about 25 nanometers) viruses. They have icosahedral T=1 capsid symmetry. They have linear single-stranded DNA genomes between 4 and 6 kilobases in length. Both ends of the genome have hairpin structures with palindromic sequences (Cotmore and Tattersall, 2014; Cotmore et al., 2014; Kailasan et al., 2015; Qiu et al., 2017). One HBoV-1 genome has been sequenced; it has a length of 5543 nucleotides (nt), and two non-identical terminal hairpins 140 and 122 nt, respectively (GenBank number JQ923422) (Kakizaki et al., 2022). Encapsulated HBoV-1 DNA strands are mostly negatively polarized (Malta et al., 2020; Shao et al., 2021). The HBoV-1 genome has two principal reading frames (Schildgen et al., 2012). The HBoV-1 genome is divided into two halves, the left (3’) half encodes non-structural NS1-4 proteins, and the right (5’) half encodes structural capsid proteins VP1-3, with VP3 as the predominant capsid protein (Schildgen et al., 2012; Malta et al., 2020; Shao et al., 2021). In the middle ORF of the genome, HBoV-1 expresses a phosphorylated non-structural protein (NP1), which overlaps with NS1 ORF at the 3’ end due to limited genomic capacity. NP1 aids in viral pre-mRNA processing and DNA replication. HBoV-1 shares transcriptional expression characteristics with other parvoviruses but also has distinct features, such as a promoter with two polyadenylation sites, (pA)p and (pA)d. HBoV-1 transcribes a single pre-mRNA from both reading frames in the negative strand (Abramczuk et al., 2015; Babkin et al., 2015; Kailasan et al., 2016; Liu et al., 2017; Malta et al., 2020; Shao et al., 2021) (**Figure 1**).

HBoV-1 encodes non-structural NS1-4 and structural capsid proteins VP1-3 on the left (3’) and right (5’) halves of its negative-strand genome, respectively, with NP1 overlapping NS1 at the 3’ end. The HBoV-1 virus generates different types of messenger RNA (mRNA) molecules through alternative splicing. One of these variants, D2-A2-spliced mRNA, contains genetic instructions that produce a protein called NS1 at the C-terminus. On the other hand, NS1-70 is generated by a different type of mRNA, referred to as D2-A2-intron-unspliced mRNA (Abramczuk et al., 2015; Babkin et al., 2015; Liu et al., 2017; Malta et al., 2020; Shao et al., 2021; Xie et al., 2022).

The HBoV-1 virus also produces additional mRNA molecules through alternative splicing from intron D1 to A1’, D1’-A1, or both. These alternative splicing events make three different mRNA variants, named R2, R3, and R4, which contain specific genetic instructions that produce three other proteins: NS2, NS3, and NS4 (Abramczuk et al., 2015; Babkin et al., 2015; Liu et al., 2017; Malta et al., 2020; Shao et al., 2021; Xie et al., 2022). These proteins play different roles in the viral life cycle, including replication, modulation of host immune response, and assembly of new viral particles. The process of alternative splicing is an essential mechanism for generating protein diversity from a limited set of genetic instructions, and it allows viruses to maximize their coding capacity and optimize their adaptation to the host environment (Abramczuk et al., 2015; Kailasan et al., 2016; Liu et al., 2017).

HBoV-1 also produces a short non-coding RNA, BocaSR, transcribed via an RNA Pol III promoter. NS1, the most extensive non-structural protein, is crucial for DNA replication, comprising N-terminal DNA-binding/endonuclease, middle helicase, and C-terminal transcription activation domains. NS1-70 lacks the C-terminus and may activate a DNA damage response like full-length NS1 (Kailasan et al., 2016; Schildgen and Schildgen, 2018; Christensen et al., 2019; Lee et al., 2019; Malta et al., 2020; Shao et al., 2021).

HBoV-1 NS2-4 function is cell type-dependent and poorly understood. In polarized human airway epithelia, HBoV-1 infection requires NS2 but not NS3 or NS4. HEK293 cells may replicate the infectious HBoV-1 duplex genome without all three. NS2 is unique to parvoviruses. NP1 and BocaSR are needed for fruitful AAV2 infection in HEK293 and HeLa cells. NS3 entirely overlaps the NS1 helicase coding area, suggesting it may act like AAV Rep52. Rep52 helps to viral genomes package. NS4 contains 199 aa and a projected 22-kDa size (Schildgen et al., 2012; Babkin et al., 2013; Schildgen, 2013; Abramczuk et al., 2015; Babkin et al., 2015; Christensen et al., 2019; Malta et al., 2020; Shao et al., 2021).

NP1 contains 200 aa and is produced from an ORF that overlaps the C-terminus of NS1. All bocaparvoviruses have it. NP1 activities are preserved among bocaparvoviruses despite a 48% amino acid sequence similarity. HBoV-1 NP1’s non-canonical nuclear localization signal is aa 7–50. NP1 boosts viral DNA replication. NP1 localized to viral DNA replication sites to compensate for the NS2 deficit during early replication, but it could not do so during late infection. NP1 processes viral pre-mRNA. NP1 is needed to define the big 3′ exon, the VP-encoding exon (Shao et al., 2021; Mattola et al., 2022).

HBoV-1 can produce three viral protein (VP) molecules, critical components of the virus’s outer shell or capsid. One of these proteins, VP2, utilizes a non-standard start codon during its production. When the virus infects cells, it synthesizes these capsid proteins in a ratio of 1:1:10, with each protein type present in the correct quantity to efficiently assemble new viral particles. VP3 is the most abundant structural component of these proteins of the capsid. VP3 plays a critical role in the assembly of virus-like particles (VLPs), non-infectious particles that mimic the virus’s structure but lack the genetic material required for replication. These VLPs include neutralizing epitopes, regions of the virus’s surface that are recognized by the immune system and stimulate the production of protective antibodies, as well as receptor binding sites, which allow the virus to attach to and enter host cells. The efficient assembly of these VLPs is essential for the virus’s ability to establish and maintain an infection. Understanding the role of HBoV-1’s capsid proteins and their interactions with host cells is crucial for developing effective antiviral therapies and vaccines (Kailasan et al., 2016; Liu et al., 2017; Ros et al., 2017; Malta et al., 2020; Fakhiri and Grimm, 2021; Shao et al., 2021; Mattola et al., 2022; Xie et al., 2022).

HBoV-1’s coding sequence features internal polyadenylation signals and a unique 90-amino acid VP1 region (VP1u), with the 11-66 amino acid segment of HBoV-1 VP1u having PLA2 activity. HBoV-1 through 4 share certain structural features, including an icosahedral fivefold axis tunnel, three trimeric protrusions, and a twofold depression. These shared features contribute to the virus’s ability to infect and replicate in host cells (Gurda et al., 2010; Kailasan et al., 2016; Liu et al., 2017; Shao et al., 2021).

Despite these similarities, the capsid shells of HBoV-1 have a flatter shape than those of other HBoV strains. This flatter shape allows HBoV-1 to package a larger viral genome with a minor VP3 protein. VP3 is an essential structural protein in the capsid that plays a critical role in protecting the virus’s genetic material and facilitating the infection of host cells. One region of the VP3 protein, known as variable surface region (VR) III, is a critical determinant of the virus’s ability to infect specific host tissues. In the case of HBoV-1, the VR III region differs from that of HBoV-2 through 4, primarily associated with gastrointestinal infections. Despite these differences, the VR III regions of HBoV-1 and HBoV-2 through 4 share a similar overall structure. Understanding the structural and functional differences between different strains of HBoV is critical for developing effective strategies for diagnosing and treating viral infections. By examining how these viruses interact with host cells and evade the immune system, researchers can gain insight into the underlying mechanisms of viral infection and identify new targets for drug and vaccine development (Babkin et al., 2013; Abramczuk et al., 2015; Liu et al., 2017; Qiu et al., 2017; Ros et al., 2017; Christensen et al., 2019; Malta et al., 2020; Bhat and Almajhdi, 2021; Shao et al., 2021; Mattola et al., 2022; Xie et al., 2022).

The HBoV-1 capsid comprises sixty primary capsid protein motifs and contains an inner core consisting of a parvovirus-typical -helix and an eight-stranded-barrel structure. These two elements combine to create the capsid, which features an open channel enclosed by a depression at the 5-fold axis, another depression at the 2-fold axes, and protrusions at the 3-fold axes. The 5-fold track is responsible for viral DNA packing, uncoating, and the externalization of VP1u. Specific variable-loop sections unique to the HBoV-1 virus are also present in the capsid and play a critical role in both infectiousness and immunogenicity. The capsid surface participates in various processes, including cell recognition, intracellular trafficking, genome packaging, host tropism, and immune response (Schildgen et al., 2012; Babkin et al., 2013; Schildgen, 2013; Abramczuk et al., 2015; Babkin et al., 2015; Principi et al., 2015; Kailasan et al., 2016; Lee et al., 2019; Malta et al., 2020; Abdelqader et al., 2021; Shao et al., 2021).

## Life cycle and pathogenesis of HBoV-1

HBoV-1 infects differentiated, polarized airway epithelia that have stopped dividing. The virus triggers the ATM, ATR, and DNA-PK pathways to facilitate its DNA replication. When the HBoV-1 genome is transfected into HEK293 cells, all three DDR pathways are activated for viral DNA replication. The NS1 protein alone can also cause DDR signaling without DNA damage. HBoV-1 genome replication in polarized human airway epithelia and HEK293 cells requires DNA repair polymerases (Christensen et al., 2019; Malta et al., 2020; Shao et al., 2021).

**Figure 2:**
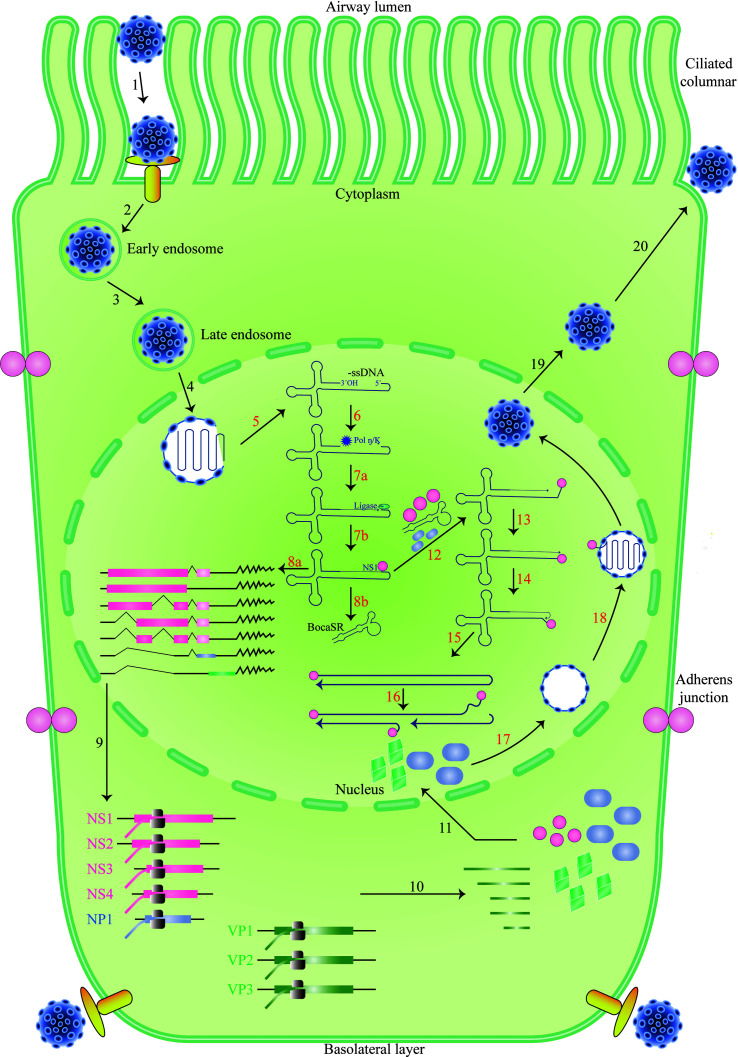
The life cycle of HBoV-1 infection. Diagrams of the cilia and junction molecules are used to represent a ciliated airway epithelial cell. The entry of HBoV-1 into cells is mediated by receptor-mediated endocytosis, followed by intracellular trafficking, and binding to an unidentified viral receptor that is expressed on both apical (ciliated) and basal cells, as illustrated (Steps 1–3). Invasion of the nucleus by the virus after it escapes from the late endosome (Step 4). The viral genome’s uncoated ssDNA is transformed into replicative form dsDNA in the nucleus, where it produces viral NS proteins and BocaSR (Steps 5–8). The viral DNA then continues to replicate in the nucleus (Steps 12–16), produces viral NS and capsid proteins (Steps 9–11), and packages its genome into an empty capsid (Steps 12–16). (Steps 16–18). Eventually, the virus developed. Based on HBoV-1 research and references from other parvoviruses, which are described in the text, the HBoV-1 infection cycle in the ciliated epithelial cell is depicted.

While HEK293 cells cannot be infected with HBoV-1, they can replicate the virus’s duplex genome, producing high titers of progeny virions upon transfection. Purified HBoV-1 virions are infectious to polarized primary human airway epithelia, cells that more closely resemble the natural host cells of the virus. When primary human airway epithelia are grown at an air-liquid interface, they can be infected by HBoV-1 through both the apical and basal surfaces. This indicates that the receptor for the virus is expressed on both ciliated apical and basal cells and that the virus may have multiple mechanisms for entering host cells (Schildgen, 2013; Broccolo et al., 2015; Schildgen and Schildgen, 2018; Christensen et al., 2019; Malta et al., 2020; Shao et al., 2021). Parvoviruses typically enter cells through a process known as receptor-mediated endocytosis, which involves the binding of viral particles to specific receptors on the cell surface, followed by internalization of the virus into the host cell. However, the particular receptor or receptors that HBoV-1 uses to enter host cells remain unknown and are an active area of research (Bhat and Almajhdi, 2021; Mattola et al., 2022). Understanding how HBoV-1 infects host cells is critical for developing effective antiviral therapies and vaccines. By identifying the specific receptor or receptors the virus uses, researchers can gain insight into the underlying mechanisms of viral infection and identify potential targets for drug development. Furthermore, studying the interactions between the virus and primary human airway epithelia can provide valuable information about the virus’s natural host range and tropism and may help to inform strategies for preventing and treating HBoV-1 infections. Upon entering the cell, the HBoV-1 virus likely traffics from early to late endosomes (Malta et al., 2020; Shao et al., 2021) (**Figure 2**).

Once it has been released in the nucleus, the viral genome is identified by the cell machinery responsible for maintaining and repairing DNA. After that, the complementary strand of the viral ssDNA genome is produced, followed by transcription and the creation of NS proteins. NS proteins are necessary for genome packing and stay connected to the viral genome throughout the replication process. The production of proteins, including BocaSR, and the replication of DNA both result from the transcription of dsDNA templates. Before entering the nucleus to be organized into capsids, the capsid proteins produced in the cytoplasm are first aggregated into oligomers. At some point in time, mature virions are discharged from the nucleus into the cytoplasm before being expelled from the cell that has been infected. A significant portion of the HBoV-1 life cycle is not yet supported by experimental evidence (Abramczuk et al., 2015; Broccolo et al., 2015; Principi et al., 2015; Qiu et al., 2017; Schildgen and Schildgen, 2018; Christensen et al., 2019; Lee et al., 2019; Bagasi et al., 2020; Malta et al., 2020; Abdelqader et al., 2021; Bhat and Almajhdi, 2021; Shao et al., 2021; Polo et al., 2022) (**Figure 2**).

Because it is challenging to culture replicative viruses using cell lines and no experimental animal models are available to simulate infection, very little research has been done on the pathogenesis of human bocaviruses. This has led to a lack of understanding of how these viruses cause human disease (Abramczuk et al., 2015; Liu et al., 2017). As a result, understanding of the disease has been limited. However, research has revealed how the HBoV-1 condition occurs in human cells, showing that HBoV-1 may modify epithelial barrier function, trigger a DNA damage response, and activate the NLRP3 inflammasome, leading to cell death through pyroptosis. Infected epithelial cells generate interleukin (IL)-1 and IL-18, contributing to bystander cell death. HBoV-1 infection also leads to increased expression of anti-apoptotic genes BIRC6 and IFI6, suggesting that it may cause chronic infection by inducing pyroptosis. HBoV-1 infections have been linked to significantly increased concentrations of interferon (IFN)-, interleukin (IL)-2, and IL-4 in patients. The activation of CD4 T cells by HBoV-1 virus-like particles increases the production of several cytokines, such as IL-10, IFN-, and IL-13. Others suggest that HBoV-1 suppresses T-helper-1 and T-helper-2 proinflammatory responses generated by rhinoviruses, even though some claim that HBoV-1 contributes to asthma exacerbations by activating T-helper-2 cytokines and proinflammatory molecules, and others claim that this contributes to asthma exacerbations (Abramczuk et al., 2015; Qiu et al., 2017; Bagasi et al., 2020; Rikhotso et al., 2020; Bhat and Almajhdi, 2021; Afumba et al., 2022; Mattola et al., 2022).

Various risk factors are associated with HBoV-1 infections, including underlying chronic medical conditions like pulmonary or cardiac disease, cancer, prematurity accompanied by chronic lung disease, and immunosuppression. These risk factors are similar to those for other respiratory viral infections. Reduced B-cell immunity may increase the chances of HBoV-1 infection because it stimulates a long-lasting IgG antibody response. HBoV-1 DNA has been detected in respiratory samples of immunocompromised individuals, and these individuals may experience a range of symptoms such as fever, lower respiratory symptoms, convulsions, encephalitis, hepatitis, and gastrointestinal symptoms. In particular, immunocompromised patients such as those undergoing chemotherapy, transplant recipients, or those with primary immunodeficiencies may be more susceptible to severe symptoms from HBoV-1 infection (De et al., 2017; Abdel-Moneim et al., 2018; Rikhotso et al., 2018; Christensen et al., 2019; Lee et al., 2019; Bagasi et al., 2020; Malta et al., 2020; Rikhotso et al., 2020; Polo et al., 2022).

However, it is essential to note that HBoV-1 DNA has also been detected in asymptomatic immunocompromised children, indicating that the virus may not always cause symptoms in these individuals. Furthermore, in one reported case, a patient exhibited prolonged DNAemia lasting over four weeks, indicating that the virus can persist in some cases. The exact mechanisms by which HBoV-1 causes disease in immunocompromised individuals are not fully understood, but it is thought to involve a complex interplay between the virus and the immune system. As with many viral infections, the severity of the disease may be influenced by factors such as the individual’s underlying health status, age, and other co-morbidities. The detection of HBoV-1 DNA in immunocompromised individuals highlights the importance of continued research into the epidemiology and pathogenesis of the virus. In particular, further studies are needed to understand better the mechanisms by which the virus interacts with the immune system and to develop effective strategies for preventing and treating HBoV-1 infections in these vulnerable populations (De et al., 2017; Abdel-Moneim et al., 2018; Rikhotso et al., 2018; Christensen et al., 2019; Lee et al., 2019; Bagasi et al., 2020; Malta et al., 2020; Rikhotso et al., 2020; Polo et al., 2022).

## Epidemiology and transmission of HBoV-1

Among several clinical studies done worldwide, HBoV-1 has been identified as a highly prevalent respiratory virus, particularly in young children, and is frequently detected in cases of respiratory tract infections, particularly those affecting children under the age of five (Kesebir et al., 2006; Lau et al., 2007; Deng et al., 2012; Koseki et al., 2012; Nokso-Koivisto et al., 2014; Zhao et al., 2014; Tabasi et al., 2016; Kenmoe et al., 2017; Rikhotso et al., 2018; Mohammadi et al., 2019a; Mohammadi et al., 2019b; Bagasi et al., 2020; Malta et al., 2020; Abdelqader et al., 2021; Polo et al., 2022). Qualitative PCR analysis of respiratory tract secretions has revealed the presence of HBoV-1 DNA in 7-25% of children with upper respiratory tract infections and 8-23% of children with lower respiratory tract infections (Christensen et al., 2019; Lee et al., 2019; Bagasi et al., 2020; Malta et al., 2020; Abdelqader et al., 2021; Polo et al., 2022). HBoV-1 DNA is present at similar levels in healthy controls. The most significant incidence of HBoV-1 DNA detection has been documented in children under 5 years old. HBoV-1 is endemic; it tends to peak in the winter and spring. It has been shown that HBoV-1 is present in less than 10% of cases of respiratory illness in adults (Christensen et al., 2019; Lee et al., 2019; Bagasi et al., 2020; Malta et al., 2020; Abdelqader et al., 2021; Polo et al., 2022).

The detection of HBoV-1 in fecal samples suggests that the virus may pass through the digestive system after being produced in the respiratory tract of children, whether or not they display respiratory symptoms (De et al., 2017; Bhat and Almajhdi, 2021). According to other reports, ninety percent of newborns in less than three months have maternal antibodies. After this age, the kid’s seropositivity continues to diminish, with no detectable maternal antibodies remaining when the infant is between six and twelve months old. Following infancy, the prevalence of HBoV rises steadily and reaches its peak when children reach the age of six, at which point between 90% and 100% of children possess antibodies against at least one of the four human bocaviruses that are circulating in their bloodstream. HBoV-1 IgG antibody concentrations remain elevated throughout adulthood, most likely due to the immune boost provided by spreading HBoV-1. According to the findings in respiratory and blood samples, no more than 20–25% of individuals who test positive for HBoV-1 DNA have an acute infection (Zaghloul, 2011; Abramczuk et al., 2015; Petrarca et al., 2016; Schildgen and Schildgen, 2018; Christensen et al., 2019; Malta et al., 2020).

DNA from HBoV-1 has almost exclusively been found in samples taken from the respiratory tract, indicating that this virus is undoubtedly spread via the respiratory system. There is no evidence that human bocaviruses may be passed down from mother to child in a vertical manner. It remains unclear whether the discovery of HBoV DNA in the plasma of healthy children and adults, including blood donors, signifies the presence of infectious HBoV viruses or merely non-infectious HBoV DNA circulating in the bloodstream. HBoV DNA has been detected in the plasma of both children and adults (Lee et al., 2019; Bagasi et al., 2020; Malta et al., 2020; Abdelqader et al., 2021; Polo et al., 2022).

## Co-infection of HBoV-1

It is not uncommon to identify more than one virus in the respiratory tracts of young infants. HBoV-1 is one of the respiratory viruses that is often identified with other such viruses. Possibly, at least one additional respiratory pathogen will be found in as much as three-quarters of the positive HBoV-1 DNA-positive respiratory samples. Some studies have shown evidence of persistent shedding of HBoV-1 for many months after the initial infection, and a third of early interactions with HBoV-1 may occur without concurrent cough or rhinorrhea. HBoV-1 is commonly found with other illnesses and may be present in asymptomatic children. However, even in cases where HBoV-1 genome is actively transcribed (mRNA is a marker of virus genome actively transcribed during an ongoing infection), nearly 60% of patients have other viruses present. Although it is challenging to study the interactions between respiratory viruses, the current research investigates the possibility of synergistic or antagonistic effects of other viruses on HBoV-1 symptomatology. Up to two years after an acute infection, there is evidence that HBoV-1 is related to a lower risk of recurrent wheezing episodes brought on by HBoV-1. This protective effect can last for up to two years (Calvo et al., 2016; Christensen et al., 2019; Malta et al., 2020; Mohammadi, 2020; Abdelqader et al., 2021; Polo et al., 2022).

## Diagnosis of HBoV-1

Qualitative PCR-based techniques that identify virus genomes in respiratory samples are often necessary to diagnose acute viral respiratory tract infections accurately (Christensen et al., 2019). However, the use of such assays is not possible when it comes to the diagnosis of HBoV-1 infections. This is because the lengthy persistence of HBoV-1 DNA in the airways makes the interpretation of a positive test result difficult (Malta et al., 2020). Compared to qualitative PCR-based tests, the specificity provided by the identification of HBoV-1-specific IgM as well as a rise or seroconversion of IgG is much greater. Because late seroconversion may occur during acute illnesses, serological assays may have limited sensitivity during these episodes (Zaghloul, 2011; Zhao et al., 2012; Abdel-Moneim et al., 2018; Malta et al., 2020). However, to achieve a conclusive diagnosis of acute HBoV-1 infection, matching blood samples must indicate either a positive IgM paired with a low IgG avidity or a 4-fold increase in the IgG titer. Both conditions must be present for the diagnosis to be considered correct. Both of these conditions must be met. Including two caveats, namely cross-reactivity and original antigenic sin, makes the serodiagnosis of HBoV-1 more complicated than it would otherwise be (Zaghloul, 2011; Abramczuk et al., 2015; Petrarca et al., 2016; Schildgen and Schildgen, 2018; Christensen et al., 2019). Studies that utilize HBoV-1 serology or mRNA detection as the reference standard have obtained more consistent outcomes and have the potential to give a basis for defining a clinical cut-off level. Acute HBoV-1 infection may be present if there are quantities of 10^4^ to 10^8^ HBoV-1 DNA copies per mL of nasopharyngeal secretions, as this has been believed to be the case. Researchers have found one hundred percent sensitivity values when using HBoV-1 mRNA as a reference for the performance of quantitative PCR (>10^6^ copies per mL) (Neske et al., 2007; Deng et al., 2012; Ghietto et al., 2012; Christensen et al., 2019; Lee et al., 2019; Malta et al., 2020; Abdelqader et al., 2021; Bhat and Almajhdi, 2021; Shao et al., 2021; Polo et al., 2022). On the other hand, specificity levels have ranged anywhere from ninety-three to ninety-nine percent. When serodiagnosis was used as the standard of comparison, the performance of quantitative PCR was as follows: the sensitivity was 81%, the specificity was 92%, and the positive predictive value (PPV) was 87%. The sensitivity and specificity refer to the diagnosis of acute infection (Christensen et al., 2019; Bagasi et al., 2020; Malta et al., 2020; Abdelqader et al., 2021; Bhat and Almajhdi, 2021; Shao et al., 2021; Polo et al., 2022). Other potentially proper diagnostic procedures include reverse transcription polymerase chain reaction, often known as RT-PCR, and immunodetection, both used to identify HBoV-1 antigen (Malta et al., 2020).

## Clinical manifestation of HBoV-1

Respiratory tract infections caused by HBoV-1 typically present clinical signs comparable to those caused by other respiratory viruses. These signs include fever, coughing, dyspnea, the common cold, rhinitis, diarrhea, vomiting, acute otitis media, pneumonia, and asthma exacerbations (Christensen et al., 2019; Lee et al., 2019; Bagasi et al., 2020; Malta et al., 2020; Polo et al., 2022). Studies based on populations with control groups have found a correlation between HBoV-1, including a high HBoV-1 load or serodiagnosis, and symptoms of upper respiratory tract infections such as coughing and rhinorrhea as acute otitis media (Midulla et al., 2010; Deng et al., 2012; Nokso-Koivisto et al., 2014; Rikhotso et al., 2018; Bagasi et al., 2020). In some od reports, reported that children who contract HBoV-1 are more likely to develop pneumonia, whereas those infected with the respiratory syncytial virus are more prone to developing bronchiolitis (Christensen et al., 2019; Malta et al., 2020; Rikhotso et al., 2020).

After respiratory syncytial virus and rhinovirus-induced bronchiolitis, HBoV-1-induced bronchiolitis comes in third place, and the illness’s severity seems comparable when age differences are considered (Midulla et al., 2010; Borchers et al., 2013; Hodinka, 2016). In some of reports, revealed that an obstructive lower respiratory tract infection was the clinical presentation seen most often. The respiratory distress caused the death of two of the youngsters. HBoV-1 has been implicated in exacerbating various respiratory disorders, including cystic fibrosis, chronic obstructive pulmonary disease (COPD), and asthma (Abramczuk et al., 2015; Broccolo et al., 2015; Schildgen and Schildgen, 2018; Christensen et al., 2019; Lee et al., 2019; Bagasi et al., 2020; Malta et al., 2020). Following an acute infection of HBoV-1, C-reactive protein concentrations and white blood cell counts typically remain within the normal range or show only slight elevation. However, chest radiography has revealed exceptions to this rule, such as the presence of infiltrates in the interstitial or peribronchial regions and hyperinflation or atelectasis. In one study, 75% of children hospitalized with lower respiratory tract infections associated with serologically confirmed HBoV-1 infections displayed interstitial infiltrates. These infections have been linked to person-to-person transmission of HBoV-1 (Manning et al., 2006; Zeng et al., 2010; Schildgen and Schildgen, 2018; Lee et al., 2019; Bagasi et al., 2020; Malta et al., 2020; Abdelqader et al., 2021; Bhat and Almajhdi, 2021; Shao et al., 2021; Polo et al., 2022).

Rarely has HBoV-1 DNA been found in respiratory samples taken from patients in controlled investigations conducted on adults and patients older than 65 years, whether those patients had or did not have an infection of the respiratory tract. DNA from the HBoV-1 virus has been found in children’s hypertrophic tonsils, adenoids, and tonsil tissue. It is still unknown whether or not these viruses have a pathogenic function in these tissues. Primary HBoV infections likely occur very seldom during pregnancy due to the high frequency of HBoV antibodies in adults. Stillborn newborns and those born with hydrops fetalis have not been shown to have HBoV infection (Hedman et al., 2010; Wang et al., 2011; Ghietto et al., 2012; Lee et al., 2019; Malta et al., 2020).

## Treatment of HBoV-1 infections

Currently, no specific therapies are licensed for HBoV infections, and there have been no comparative trials on antiviral medications. A *post-hoc* analysis of a randomized controlled trial on wheezing children with serologically confirmed HBoV-1 infection showed that prednisolone was ineffective in treating the symptoms. Therefore, supportive care is the preferred therapy for children with severe bronchiolitis, wheezing, or pneumonia, with bronchodilation and respiratory support being the most significant types. While HBoV-1 has been associated with acute respiratory infections, the illness is usually self-limiting and does not cause complications. There are no specific preventive measures that can be taken (Abramczuk et al., 2015; Rikhotso et al., 2018; Christensen et al., 2019).

## Conclusion

HBoV-1 is now considered to be one of the important pathogenic factors of acute respiratory infections in children. HBoV-1 contains more information than other bocaviruses in humans. Most research has discovered HBoV-1 in respiratory tract secretions using PCR, although few have confirmed its acute infection using more accurate techniques. Future progress hinges on more robust diagnostic criteria. To identify acute primary HBoV-1 infection, at least two measures must be present: high DNA load in nasopharyngeal secretions, mRNA, positive IgM, or a 4-fold increase in IgG titer in matching blood tests.

## Author contributions

The author confirms being the sole contributor of this work and has approved it for publication.
